# Ankle-Brachial Index Is Independently Associated With Cardiovascular Outcomes and Foot Ulcers in Asian Patients With Type 2 Diabetes Mellitus

**DOI:** 10.3389/fendo.2021.752995

**Published:** 2021-11-18

**Authors:** Ming-Chi Yang, Yu-Yao Huang, Sheng-Hwu Hsieh, Jui-Hung Sun, Chih-Ching Wang, Chia-Hung Lin

**Affiliations:** ^1^ Division of Endocrinology and Metabolism, Department of Internal Medicine, Chang Gung Memorial Hospital, Linkou, Taiwan; ^2^ College of Medicine, Chang Gung University, Taoyuan, Taiwan; ^3^ Department of Anesthesiology, Kaohsiung Chang Gung Memorial Hospital, Kaohsiung, Taiwan

**Keywords:** diabetic foot, peripheral arterial disease, ankle-brachial index (ABI), type 2 diabetes mellitus, cardiovascular complications

## Abstract

**Background and Aims:**

The ankle-brachial index (ABI) is an efficient tool for objectively documenting the presence of lower-extremity peripheral arterial disease (PAD). The predictive factors of cardiovascular events and diabetic foot ulcer were not clear from the ABI examination in Taiwanese patients with type 2 diabetes mellitus (DM).

**Methods:**

We enrolled 482 patients with type 2 DM who regularly visited the outpatient department of Chang Gung Memorial Hospital and received ABI as well as brachial-ankle pulse wave velocity (ba-PWV) examinations from 2010 to 2017. Age, gender, PAD symptoms, comorbidities, family history of chronic diseases, lifestyle (smoking, alcohol consumption, and exercise), height, weight, waist circumference, monofilament testing and foot ulcer status were studied.

**Results:**

There were 104 (22%) patients (mean age, 67.8 years) with the ABI <1.0. These patients with low ABI (ABI<1.0) had a significantly older age (p=0.001), higher delta PWV (p<0.001), higher rates of stroke (p=0.007), myocardial infarction (p=0.016), and foot ulcer (p=0.039). In a multivariable analysis model, the adjusted odds ratio (aOR) for myocardial infarction, stroke, and foot ulcers associated with low ABI were 1.219 (0.397-3.743, p=0.729), 1.204 (0.556-2.610, p=0.638), and 2.712 (1.199-6.133, p=0.017), respectively. The patients with low PWV (PWV<1400 cm/s) were significantly younger (p<0.001) and had a lower rate of hypertension (p<0.001), and higher percentages of stroke (p=0.027) and dialysis (p=0.041) family history.

**Conclusions:**

Low ABI was associated with cardiovascular events and diabetic foot ulcer independently in patients with type 2 DM.

## Introduction

Diabetic foot ulcer is a common problem for patients with diabetes mellitus (DM). It occurs as a result of various factors, such as mechanical changes in conformation of the bony architecture of the foot, peripheral neuropathy, and atherosclerotic peripheral arterial disease, all of which occur with higher frequency and intensity in the diabetic population (1).

Risk of cardiovascular disease is also known to be higher in patients with DM. It is related in part to insulin resistance, which causes lipid profile abnormalities affecting the vascular system (2, 3).

The ankle-brachial index (ABI) is an efficient tool for objectively documenting the presence of lower-extremity peripheral arterial disease (PAD). It is a simple, reproducible, and cost-effective assessment that can be used to detect lower-extremity arterial stenosis in the primary care setting. In patients with DM, ABI is often checked to assess the vascular risk for PAD (1).

However, predictive factors for cardiovascular events and diabetic foot ulcer during the ABI examination were not clear for Taiwanese patients with type 2 DM. The purpose of this study is to determine predictive factors for the occurrence of diabetic foot ulcer and cardiovascular events in the ABI examination.

## Patients and Methods

### Patient Enrollment

We enrolled 482 patients with type 2 DM who regularly visited the outpatient department of Chang Gung Memorial Hospital and underwent ABI and brachial-ankle pulse wave velocity (ba-PWV) examinations from 2010 to 2017. The inclusion criteria included the patients with type 2 DM and completed the exam with regular follow-up as well as comprehensive diabetic complication records. The exclusion criteria included those who lack of completely related diabetic complication records. The study was approved by the Institutional Review Board of Chang Gung Memorial Hospital, Linkou, Taiwan.

### Measurement of PAD Indexes (ABI and ba-PWV)

The ABI and ba-PWV were measured using the Vascular Profiler 1000 (Colin Co. Ltd., Komaki, Aichi, Japan), while patients were in the supine position and after resting for at least 5 minutes in an air-conditioned room (approximately 25°C). Systolic blood pressure (SBP), diastolic blood pressure (DBP), ba-PWV, and electrocardiogram data were recorded simultaneously. The equation used to calculate ba-PWV is as follows: ba-PWV = (La − Lb)/Tba, where La represents the length from the suprasternal notch to the ankle (La = 0.8129 × height (cm) + 12.328), Lb represents the length from the suprasternal notch to the right brachium (Lb = 0.2195 × height (cm) − 2.0734), and Tba is the time interval between the brachial and ankle arteries measured between the front wave of the brachial and ankle waveforms. In this study, we chose the larger ba-PWV value among one patient’s right and left lower extremities for statistical analysis, and we defined delta-PWV as the absolute difference between one patient’s right and left PWV value. The ABI was determined as the ratio of ankle SBP to brachial SBP. The same operator performed all examinations during the investigation period; thus, the operator-related variability was low.

### Clinical Examination

The patients’ clinical data were obtained *via* a questionnaire. Questionnaire items included age, gender, PAD symptoms, history of chronic disease, family history of chronic disease, lifestyle (smoking, alcohol consumption, and exercise), height, weight, waist circumference, monofilament testing, dorsalis pedis artery exam, and foot ulcer status.

### Definition of Low ABI

In general, a normal ABI value is considered to be between 1.0-1.4. A value above 1.4 suggests a non-compressible calcified vessel. A value below 1.0 suggests PAD. In this study, we separated the enrolled patients into two groups, using the cut point of ABI=1.0 and compared the clinical data and ba-PWV between them.

### Statistical Analysis

Statistical analyses were performed using independent sample *t*-test to detect differences in age, height, weight, waist circumference, body mass index (BMI), ba-PWV, and delta PWV between the two groups of patients (ABI≥1.0 and ABI<1.0), and to detect differences in age, height, weight, waist circumference, BMI and ABI between the two groups of patients (PWV≥1400 and PWV<1400 cm/s). Chi-square tests were used to assess differences in gender, smoking status, alcohol consumption, exercise frequency, myocardial infarction history, hypertension history, stroke history, dialysis history, family history of DM, family history of hypertension, family history of myocardial infarction, family history of stroke, family history of dialysis, monofilament testing, and foot ulcer between the two groups of patients (ABI≥1.0 and ABI<1.0). If the sample sizes were small, we used Fisher’s exact test to perform statistical analyses. The adjusted odds ratios (aOR) of low ABI for myocardial infarction, stroke, and foot ulcer were determined by multivariate logistic regression after adjusting for potential confounders. Statistical significance was defined as p value<0.05. All statistical analyses were performed using the Statistical Package for the Social Sciences (SPSS) version 19.0 for Windows (SPSS Inc., Chicago, IL, USA).

## Results

A total of 482 patients with type 2 DM were enrolled in our study. Patients had a mean age of 67 years (256 men, 226 women). Patients were divided into two groups depending on their ABI value (ABI≥1.0 and ABI<1.0). The ABI value of 378 patients (78%) was ≥1.0, while the other 104 patients (22%) had an ABI<1.0. **Table 1** showed that patients with low ABI (ABI<1.0) had a significantly higher age (p=0.001) and higher delta PWV (p<0.001). Also, patients with low ABI had a significantly higher rate of stroke (p=0.007), myocardial infarction (p=0.016), and foot ulcer (p=0.039).

**Table 1:** Variables in patients with low ABI (<1) *versus* patients with normal ABI (≧1).

	All (n=482)	ABI<1.0 (n=104)	ABI≧1.0 (n=378)	p value
Age (years)	64.6 ± 10.9	67.8 ± 11.3	63.7 ± 10.7	0.001*
Height(cm)	159.6 ± 8.7	159.4 ± 9.0	159.7 ± 8.6	0.714
Weight(kg)	65.5 ± 11.7	64.5 ± 11.2	65.7 ± 11.9	0.366
Waist circumference (cm)	88.2 ± 9.2	89.4 ± 8.8	87.9 ± 9.3	0.16
BMI(kg/m^2^)	25.6 ± 3.7	25.3 ± 3.5	25.7 ± 3.8	0.363
ba-PWV(cm/s)	1794.0 ± 411.3	1837.1 ± 543.9	1782.2 ± 366.5	0.333
delta PWV(cm/s)	121.2 ± 267.7	253.7 ± 440.8	85.8 ± 182.6	<0.001*
Male	53.1%	58.7%	51.6%	0.223
Soreness of upper extremity	48.3%	48.1%	48.4%	1.000
Soreness of lower extremity	63.7%	71.2%	61.6%	0.084
Pain of upper extremity	19.1%	15.4%	20.1%	0.325
Pain of lower extremity	35.7%	43.3%	33.6%	0.083
Intermittent claudication	5%	4.8%	5.0%	1.000
Coldness of upper extremity	38.2%	42.3%	37.0%	0.362
Coldness of lower extremity	48.1%	53.8%	46.6%	0.223
MI	6.9%	12.6%	5.4%	0.016*
HTN	66.4%	72.1%	64.8%	0.197
Dialysis	1.5%	1.9%	1.3%	0.647
Stroke	12.9%	21.2%	10.6%	0.007*
Smoking	13.9%	12.6%	14.3%	0.750
Alcohol	22.5%	20.6%	23.0%	0.689
Exercise	80%	73.7%	81.6%	0.090
Family history of DM	63.9%	60.4%	64.8%	0.464
Family history of HTN	46.5%	44.9%	47.0%	0.811
Family history of MI	6.5%	5.4%	6.7%	0.813
Family history of stroke	25.2%	27.8%	24.5%	0.512
Family history of dialysis	6.3%	4.2%	6.9%	0.478
Abnormal monofilament testing	4.7%	8.2%	3.7%	0.098
Foot ulcer	8.2%	13.6%	6.6%	0.039*

BMI, body mass index (kg/m^2^); ba-PWV, brachial-ankle pulse wave velocity (m/s); delta PWV, the absolute difference between one patient’s right and left PWV value; MI, myocardial infarction; DM, diabetes mellitus; HTN, hypertension.

Date presented as mean ± standard deviation or %. *p < 0.05 by independent sample t-test for continuous variables and Chi-square or Fisher’s exact tests as indicated for nominal variables.

**Tables 2**–**4** showed aORs (95% confidence intervals) of low ABI for myocardial infarction, stroke, and foot ulcer respectively. Three models were built after adjusting for potential confounders, including gender, age, BMI, smoking, alcohol consumption, exercise, family history of DM, family history of hypertension, family history of myocardial infarction, family history of stroke, and family history of dialysis. In model 1, gender, age, and BMI were adjusted. The aOR for myocardial infarction, stroke, and foot ulcer of low ABI were 2.065 (0.965-4.419, p=0.062), 1.994 (1.100-3.618, p=0.023) and 2.567 (1.243-5.302, p=0.011) respectively. In model 2, gender, age, BMI, smoking, and alcohol consumption were adjusted. The aOR for myocardial infarction, stroke, and foot ulcer of low ABI were 2.114 (0.972-4.597, p=0.059), 1.899 (1.036-3.483, p=0.038) and 2.628 (1.269-5.441, p=0.009) respectively. In model 3, gender, age, BMI, smoking, alcohol consumption, exercise, family history of DM, family history of hypertension, family history of myocardial infarction, family history of stroke, and family history of dialysis were adjusted. The aOR for myocardial infarction, stroke, and foot ulcer of low ABI were 1.219 (0.397-3.743, p=0.729), 1.204 (0.556-2.610, p=0.638) and 2.712 (1.199-6.133, p=0.017) respectively.

**Table 2:** Odds ratios (OR) for myocardial infarction by logistic regression with multivariable models.

	Model 1			Model 2			Model 3		
	OR	CI 95%	p value	OR	CI 95%	p value	OR	CI 95%	p value
Sex (Male)	3.554	1.525,8.284	0.003*	4.920	2.091,11.574	<0.001*	10.706	2.976,38.513	<0.001*
Age (years)	1.065	1.023,1.109	0.002*	1.048	1.007,1.091	0.022*	1.029	0.974,1.088	0.309
BMI (kg/m^2^)	1.054	0.953,1.167	0.308	1.060	0.958,1.172	0.262	1.035	0.912,1.175	0.595
Low ABI	2.065	0.965,4.419	0.062	2.114	0.972, 4.597	0.059	1.219	0.397,3.743	0.729
Smoking				0.000	0.000	0.997	0.000	0.000	0.997
Alcohol				0.409	0.133,1.255	0.118	0.135	0.017,1.080	0.059
Exercise							0.682	0.213,2.177	0.518
DM family history							0.340	0.112,1.034	0.057
HTN family history							1.154	0.392,3.394	0.795
MI family history							5.985	1.431,25.024	0.014*
Stroke family history							1.177	0.361,3.839	0.787
Dialysis family history							0.000	0.000	0.998

BMI, body mass index (kg/m^2^); Low ABI, ankle-brachial index < 1.0; DM, diabetes mellitus; HTN, hypertension; MI, myocardial infarction; CI, confidence intervals.

*p < 0.05 by multivariate logistic regression.

**Table 3:** Odds ratios (OR) for stroke by logistic regression with multivariable models.

	Model 1			Model 2			Model 3		
	OR	CI 95%	p value	OR	CI 95%	p value	OR	CI 95%	p value
Sex	1.376	0.784,2.415	0.267	1.770	0.980,3.198	0.058	1.304	0.649,2.619	0.456
Age (years)	1.045	1.016,1.076	0.002*	1.037	1.008, 1.067	0.013*	1.048	1.011,1.087	0.010*
BMI (kg/m^2^)	1.106	1.028,1.189	0.007*	1.108	1.031,1.191	0.005*	1.069	0.985,1.160	0.112
Low ABI	1.994	1.100,3.618	0.023*	1.899	1.036,3.483	0.038*	1.204	0.556,2.610	0.638
Smoking				0.460	0.150,1.405	0.173	0.610	0.182,2.047	0.424
Alcohol				0.602	0.273,1.326	0.208	0.726	0.287,1.841	0.500
Exercise							0.427	0.206,0.884	0.022*
DM family history							1.126	0.547,2.317	0.748
HTN family history							1.047	0.506,2.168	0.901
MI family history							0.715	0.178,2.865	0.635
Stroke family history							2.413	1.147,5.077	0.020*
Dialysis family history							0.963	0.193,4.795	0.963

BMI, body mass index (kg/m^2^); Low ABI, ankle-brachial index < 1.0; DM, diabetes mellitus; HTN, hypertension; MI, myocardial infarction; CI, confidence intervals.

*p < 0.05 by multivariate logistic regression.

**Table 4:** Odds ratios (OR) for foot ulcer by logistic regression with multivariable models.

	Model 1			Model 2			Model 3		
	OR	CI 95%	p value	OR	CI 95%	p value	OR	CI 95%	p value
Sex	1.329	0.657,2.687	0.429	1.182	0.546,2.557	0.671	1.517	0.621,3.705	0.360
Age (years)	0.961	0.933,0.989	0.008*	0.964	0.935,0.994	0.018*	0.965	0.930,1.000	0.050
BMI (kg/m^2^)	0.998	0.911,1.093	0.959	0.995	0.908,1.091	0.916	0.988	0.891,1.096	0.820
Low ABI	2.567	1.243,5.302	0.011*	2.628	1.269,5.441	0.009*	2.712	1.199,6.133	0.017*
Smoking				1.542	0.625,3.801	0.347	1.573	0.563,4.398	0.388
Alcohol				1.091	0.487,2.445	0.832	0.803	0.301,2.144	0.662
Exercise							0.504	0.209,1.219	0.128
DM family history							0.805	0.351,1.846	0.608
HTN family history							0.983	0.421,2.295	0.969
MI family history							0.920	0.170,4.977	0.923
Stroke family history							1.070	0.406,2.816	0.891
Dialysis family history							0.628	0.073,5.414	0.672

BMI, body mass index (kg/m^2^); Low ABI, ankle-brachial index < 1.0; DM, diabetes mellitus; HTN, hypertension; MI, myocardial infarction; CI, confidence intervals.

*p < 0.05 by multivariate logistic regression.

**Table 5** shows that patients with low PWV (PWV<1400 cm/s) had a significantly lower age (p<0.001). Also, patients with low PWV had a significantly lower rate of hypertension (p<0.001). However, patients with low PWV had a significantly higher rate of upper limb soreness (p=0.032), family history of stroke (p=0.027), and family history of dialysis (p=0.041).

**Table 5:** Variables in patients with high PWV (PWV≧1400 m/s) *versus* patients with normal PWV (PWV<1400 m/s).

	PWV＜1400 cm/s (n=64)	PWV≧1400 cm/s (n=418)	p value
Age (years)	55.4 ± 11.5	66.0 ± 10.2	<0.001*
Height (cm)	161.3 ± 9.4	159.4 ± 8.6	0.109
Weight (kg)	68.5 ± 14.8	65.0 ± 11.1	0.076
Waist circumference (cm)	88.0 ± 11.5	88.3 ± 8.8	0.845
BMI (kg/m^2^)	26.2 ± 4.5	25.5 ± 3.6	0.172
ABI	1.01 ± 0.16	1.05 ± 0.14	0.103
Male	53.1%	53.1%	1.000
Soreness of upper extremity	60.9%	46.4%	0.032*
Soreness of lower extremity	68.8%	62.9%	0.405
Pain of upper extremity	25.0%	18.2%	0.231
Pain of lower extremity	42.2%	34.7%	0.264
Intermittent claudication	7.8%	4.5%	0.348
Coldness of upper extremity	35.9%	38.5%	0.783
Coldness of lower extremity	54.7%	47.1%	0.284
MI	1.6%	7.8%	0.106
HTN	43.8%	69.9%	<0.001*
Stroke	10.9%	13.2%	0.841
Dialysis	1.6%	1.4%	0.621
Smoking	19.0%	13.2%	0.240
Alcohol	28.6%	21.5%	0.256
Exercise	79.0%	80.1%	0.865
Family history of DM	61.0%	64.3%	0.663
Family history of HTN	50.9%	45.8%	0.480
Family history of MI	8.3%	6.1%	0.568
Family history of stroke	37.1%	23.3%	0.027*
Family history of dialysis	13.1%	5.3%	0.041*
Abnormal monofilament testing	0.0%	5.4%	0.093
Foot ulcer	9.8%	7.9%	0.616

PWV, pulse wave velocity (cm/s); BMI, body mass index (kg/m^2^); ABI, ankle-brachial index; MI, myocardial infarction; DM, diabetes mellitus; HTN, hypertension.

Date presented as mean ± standard deviation or %. *p < 0.05 by independent sample t-test for continuous variables and Chi-square or Fisher’s exact tests as indicated for nominal variables.

## Discussion

The major finding of the present study was that patients with low ABI had a significantly higher rate of stroke, myocardial infarction, and foot ulcer.

### Relationship Between Low ABI and Stroke

In the current study, diabetic patients with low ABI had a significantly higher rate of stroke (p=0.007). A meta-analysis conducted by Hongjie Fan and colleagues demonstrated that a low ABI appears to be an independent predictor for ischemic and recurrent stroke events at the age range of 55-93 years (4). One study demonstrated that low ABI is associated with risk of stroke or transient ischemic attack in a group of 251 men and 423 women with a mean age of 80 years (5). Another study showed that low ABI appears to be associated with a higher risk of early recurrent stroke in patients with acute cerebral ischemia and no history of symptomatic PAD at mean age 64 ± 14 years (6). A Korean study showed that low ABI is linearly associated with large artery stenosis, but not the entire spectrum of ischemic stroke mechanism at patients with mean age of 65 ± 10 years (7). However, this study also revealed a strong correlation between small vessel disease and ba-PWV, and between overall ischemic stroke and ba-PWV. This phenomenon was not found in our current study. One study by Barreto-Neto N. and colleagues demonstrated that ABI is independently associated with intracranial atherosclerotic stenosis in a sample of patients with mean age 62 ± 15 years and ischemic stroke (8). One meta-analysis study showed that in patients at the mean age ranged from 64 to 79 years with acute ischemic stroke, low ABI increased the risk of composite outcomes (myocardial infarction, stroke, or mortality), disability, and mortality (9). The significance of the current study demonstrated that even at a younger age (around 60 years old), patients with diabetes have a higher risk of stroke along with lower ABI levels.

### Relationship Between Low ABI and Myocardial Infarction

In the current study, patients with low ABI had a significantly higher rate of myocardial infarction (p=0.016). A meta-analysis conducted by Hao Z. and colleagues showed that low ABI increased the risk of composite outcomes (myocardial infarction, stroke, or mortality), disability, and mortality in patients with acute ischemic stroke (9). Another meta-analysis conducted by Fowkes FG and colleagues has demonstrated that measurement of the ABI may improve the accuracy of predicting cardiovascular risk (10). A study of 3627 patients with chronic kidney disease showed that ABI<1.0 was related to risk of PAD, myocardial infarction, and composite cardiovascular disease (11). A study of 5248 individuals showed inclusion of the ABI improves the predictive capacity of the Framingham-REGICOR risk function. The study results indicate the potential value of including this simple test in cardiovascular risk stratification and supports current guidelines and recommendations (12). One Japanese study showed that ABI, hypertension, estimated glomerular filtration rate, max-IMT (thickest points for the intima-media thickness in the common carotid arteries, carotid bulb, and internal carotid arteries) and use of diabetic medication were independent predictive factors for coronary artery stenosis in patients with type 2 DM, and the authors built a novel formula to predict coronary stenosis in type 2 DM patients using these five factors (13). A study of 375 patients with acute myocardial infarction showed ABI is an important predictor of polyvascular disease and is a useful and simple measurement that appeared to be predictive in widespread atherosclerosis among these patients (14). Among subjects with abnormal ABI, the risk of hospitalization for myocardial infarction was higher in those with previous cardiovascular events (15). Compared with high ABI, the occurrence rate of myocardial infarction could be as high as 5% in those with low ABI at the median follow-up of 30 months (16). Therefore, those diabetic patients with low ABI should be monitored closely in the early prevention of myocardial infarction.

### Relationship Between Low ABI and Foot Ulcer Status

In the current study, patients with low ABI had a significantly higher rate of having foot ulcer (p=0.039). A Korean study of 126 diabetic patients who were consecutively enrolled showed that checking the ABI by office screening was a simple technique for evaluating pathologic change in the diabetic foot, and that this screening, based on treatment-oriented classification, helped to reduce pedal complications in a diabetic population (17). A systemic review conducted by Brownrigg and colleagues showed that among patients with diabetic foot ulcer, ABI <0.5 is associated with a significant increase in the incidence of major amputation (18). The significance of the current study further demonstrates the predictive power of low ABI in diabetic foot ulcer even with the adjustments of common baseline variables in patients with type 2 DM in Taiwan.

### Relationship Between High PWV, Age, and Hypertension

In the current study, patients with high PWV had a significantly older age (p<0.001) and higher rate of hypertension history (p<0.001). A study conducted by Smulyan and colleagues has demonstrated that increased arterial stiffness is an important, independent, and significant risk factor in subjects with hypertension and diabetes (19). An article written by Munakata mentioned that PWV could be a global cardiovascular marker, since it increases with advancing age, high blood pressure, hyperglycemia, and other cardiovascular risk factors. A 1 cm/s increase in ba-PWV is associated with a 12% increase in the risk of cardiovascular events (20). In the current study, a high PWV did not correlate with the severity of symptoms in patients with diabetes. In addition, the symptoms of PAD were even mild in patients with high PWV. This finding showed the possibility of unawareness of nerve stimulation and the severe squeals in the diabetic patients with high PWV. The high PWV was associated with reduced arterial flow volume in patients with type 2 diabetes (21).We should pay more attention to those diabetic patients who are elderly and have a history of hypertension.

### Limitations

The main limitation of our study is that the numbers in each group were not even. Although this is a simple outpatient measurement, it is still regulated by the authority of National Health Insurance in Taiwan. If the patient had no symptoms or signs of peripheral artery disease, the exam was not covered by the insurance. However, the significant findings were still solid and comparable with previous studies. Nevertheless, this is the first and largest cohort observation study in patients with type 2 DM in Taiwan. Ethnic differences were not elaborated in this study.

In conclusion, our results demonstrated that values of ABI were associated with stroke, myocardial infarction and independently with diabetic foot in patients with type 2 DM. Screening patients with type 2 DM using ABI, a simple and noninvasive marker, may help identify patients at high risk of cardiovascular events and foot ulcer.

## Data Availability Statement

The original contributions presented in the study are included in the article/supplementary material. Further inquiries can be directed to the corresponding author.

## Ethics Statement

The study was approved by the Institutional Review Board of Chang Gung Memorial Hospital, Linkou, Taiwan. The patients/participants provided their written informed consent to participate in this study.

## Author Contributions

M-CY. wrote the manuscript and researched data. S-HH researched data and contributed to discussion. Y-YH contributed to discussion and reviewed/edited the manuscript. J-HS researched data. C-CW and C-HL reviewed/edited the manuscript. C-CW and C-HL are the guarantors of this work, have full access to all the data in the study, and take responsibility for the integrity of the data and the accuracy of the data analysis. All authors contributed to the article and approved the submitted version.

## Funding

This work was funded by a grant (CMRPG3H0401, CMRPG3H0402, CMRPG3H0403, CMRPG3H0941, CMRPG3H0942 CMRPG3H0943) from Chang Gung Memorial Hospital and a grant (MOST 105-2628-B-182A-007-MY3, MOST 109-2314-B-182 -049 -MY3) from Ministry of Science and Technology, R.O.C. The funders had no input into any aspect of the design and management of this study. The authors declare no conflict of interest.

## Conflict of Interest

The authors declare that the research was conducted in the absence of any commercial or financial relationships that could be construed as a potential conflict of interest.

## Publisher’s Note

All claims expressed in this article are solely those of the authors and do not necessarily represent those of their affiliated organizations, or those of the publisher, the editors and the reviewers. Any product that may be evaluated in this article, or claim that may be made by its manufacturer, is not guaranteed or endorsed by the publisher.
