# Corrigendum: Ankle-Brachial Index Is Independently Associated With Cardiovascular Outcomes and Foot Ulcers in Asian Patients With Type 2 Diabetes Mellitus

**DOI:** 10.3389/fendo.2022.878492

**Published:** 2022-03-16

**Authors:** Ming-Chi Yang, Yu-Yao Huang, Sheng-Hwu Hsieh, Jui-Hung Sun, Chih-Ching Wang, Chia-Hung Lin

**Affiliations:** ^1^ Division of Endocrinology and Metabolism, Department of Internal Medicine, Chang Gung Memorial Hospital, Linkou, Taiwan; ^2^ College of Medicine, Chang Gung University, Taoyuan, Taiwan; ^3^ Department of Anesthesiology, Kaohsiung Chang Gung Memorial Hospital, Kaohsiung, Taiwan

**Keywords:** diabetic foot, peripheral arterial disease, ankle-brachial index (ABI), type 2 diabetes mellitus, cardiovascular complications

In the originally published article, two affiliations for the first author Ming-Chi Yang were omitted. In addition to the “Division of Endocrinology and Metabolism, Department of Internal Medicine, Chang Gung Memorial Hospital, Linkou, Taiwan”, the article should also have indicated that this author was affiliated with the “College of Medicine, Chang Gung University, Taoyuan, Taiwan” and the “Department of Anesthesiology, Kaohsiung Chang Gung Memorial Hospital, Kaohsiung, Taiwan”.

The authors apologize for this error and state that this does not change the scientific conclusions of the article in any way. The original article has been updated.

## Publisher’s Note

All claims expressed in this article are solely those of the authors and do not necessarily represent those of their affiliated organizations, or those of the publisher, the editors and the reviewers. Any product that may be evaluated in this article, or claim that may be made by its manufacturer, is not guaranteed or endorsed by the publisher.

